# Broadband stripline Lenz lens achieves 11 × NMR signal enhancement

**DOI:** 10.1038/s41598-023-50616-0

**Published:** 2024-01-18

**Authors:** Jianyi Liang, Hossein Davoodi, Sagar Wadhwa, Vlad Badilita, Jan G. Korvink

**Affiliations:** 1 Institute of Microstructure Technology (IMT), Karlsruhe Institute of Technology (KIT), 76344 Eggenstein-Leopoldshafen, Germany; 2 Present Address: Littelfuse Inc., 68623 Lampertheim, Germany; 3 Present Address: Voxalytic GmbH, 76228 Karlsruhe, Germany

**Keywords:** Electrical and electronic engineering, Solution-state NMR

## Abstract

A Lenz lens is an electrically passive conductive element that, when placed in a time-varying magnetic field, acts as a magnetic flux concentrator or a magnetic lens. In the realm of nuclear magnetic resonance (NMR), Lenz lenses have been exploited as electrically passive metallic radiofrequency interposers placed between a sample and a tuned or untuned NMR detector in order to focus the ${B}_1$-field of the detector onto a smaller sample space. Here we explore a novel embodiment of the Lenz lens, which acts as a non-resonant stripline interposer, i.e., the ${B}_1$-field acts along the longitudinal volume of a sample container, such as a capillary or other microfluidic channel that is coincident with the axis of the stripline. The almost vanishing self-resonance of the stripline Lenz lens, at frequencies relevant for NMR, leads to a desirable ${B}_1$-field amplitude that is nearly perfectly uniform across the sample and hence lacking a characteristic sinusoidal modal shape. The action of Lenz’ law ensures that no stray ${B}_1$-field is found outside of the stripline’s active volume. Because the stripline Lenz lens does not rely on its own geometry to achieve resonance, its frequency response is thus widely broadband for field enhancements up to a factor of 11, with only the external driving resonator properties governing the overall resonant behaviour. We explore the use of the stripline Lenz lens with a sub-nanolitre sample volume, readily detecting 4 isotopes with resonances ranging from 125.76 to 500 MHz. The concept holds potential for the NMR study of thin films, small biological samples, as well as the in situ study of battery materials.

## Introduction

Nuclear magnetic resonance (NMR) is a non-invasive and non-destructive analytic technique that interrogates atomic nuclei and their molecular environment via radio frequency (RF) radiation. This reveals an exquisitely detailed inner picture for a wide range of materials, providing insight into their structures and dynamics with atomic level resolution. NMR also suffers from low sensitivity. In a field of 11.74 T, roughly only 1 in about 100000 identical spins contributes to the total detected NMR signal. The equation describing the corresponding signal-to-noise ratio (SNR) suggests several ways to overcome this issue^1^.

The general yet *brute force* method to obtain more sensitivity resorts to exposing a sample to an ever higher static $B_0$ magnetic field. For this expensive option, SNR scales with $B_0^{7/4}$. Spin hyperpolarization is more effective, raising polarisation by several orders of magnitude, but is limited in generality, since experimental conditions have to closely match sample requirements. A lower temperature can increase sample polarisation, and decrease equipment-introduced noise, hence actively cooled detectors are routinely used in high resolution NMR spectroscopy. Nevertheless, not all samples and processes can be subjected to arbitrarily low temperatures.

Miniaturised NMR detectors are advantageous when the amount of sample is limited, and arise in numerous practical situations, such as working with toxic, expensive, or otherwise limited-availability samples, or the necessity to perform a large number of experiments and reduce the associated waste. The smallest possible NMR detector that fully contains the sample to be analyzed, leads to the highest achievable SNR^2^.

Yet the miniaturization of NMR detectors also poses a series of challenges^3^. Usually, miniaturization implies a dense arrangement of constituent materials in a very limited volume, e.g., metals for the detector itself, glass and/or polymers as substrate and sample holder, as well as the sample itself. The materials may exhibit very different magnetic susceptibilities, which lead to $B_0$ non-uniformities degrading both spectral resolution and SNR. $B_1$ uniformity in the limited sensitive volume of a miniaturized NMR detector requires precise RF field optimization. Sample handling at small length scales poses its own specific challenges as well.

The literature reports numerous variants of miniaturized detectors. The first report featured hand-wound solenoids around capillaries^2^. Planar loop or spiral coils were explored early on, relying on planar microfabrication techniques^4–8^. In spite of relatively poor $B_1$ uniformity, the simplicity of integration with microfluidic channels facilitated sample handling^9–13^. Other variants included Helmholtz coils and planar phased arrays^14,15^. At the nanometer scale, solutions involving magnetic resonance force microscopy and NV-centers have contributed to a paradigm shift^16–18^, and even opening up new horizons in the field of miniaturised quantum sensors.

As an additional strategy, striplines have resolved a number of challenges brought by miniaturization: affordable fabrication that allows for batch manufacturing; their slender predominant dimension can be easily aligned to the static field, thus not only inherently creating a $B_1$ field perpendicular to the static field, but also minimizing the $B_0$ jumps and offering an intrinsically good spectral resolution. Even early approaches have reported sensitivities better than 0.5 nmol/ $\sqrt {{\text{Hz}}}$, $B_1$ homogeneity ($A_{810}/A_{90}$) better than 76%, and sub-Hz spectral resolution^19–21^. Stripline NMR detectors have demonstrated their versatility in handling both liquid and planar thin film samples, being employed for in-situ NMR monitoring of chemical reactions^22^ or metabolomic analysis^23,24^, continuous flow measurements with flow rates between 0.55 μL/min and 15 μL/min^25^, as well as together with generic DNP enhancement of solid state ${}^{1}$H factors of up to 500 for H$_{2}$O/D$_{2}$O/d$_{6}$-glycerol samples doped with TEMPOL radicals^26^.

Remarkably, the beautifully simple structure of stripline detectors has proven to be amenable to integration with a series of add-ons, offering additional capabilities: a tapered stripline structure is generating arbitrarily-defined one dimensional $B_1$ gradients enabling spatially resolved spectroscopy even in inhomogeneous static fields^27^, or acting as a simple and cheap alternative to $B_0$ pulsed field gradients^28^. An innovative combination between a stripline (for detection) and a flattened solenoid-like coil (for transmission) was employed to demonstrate novel differential-mode NMR measurements^29^.

In this work, borrowing from another branch of miniaturised NMR detectors, i.e., the Lenz lens, we developed a stripline that is wirelessly connected to the spectrometer hardware. Lenz lenses were previously described as a method to manipulate the magnetic flux distribution by controlling the induced currents in passive elements^30^, eventually being studied, optimised and introduced in the design of NMR detectors^31–34^. Lenz lens structures collect and concentrate the magnetic flux in a certain volume and are intrinsically broadband^35^. By integrating the stripline detector with the Lenz lens add-on in a novel structure called stripline Lenz lens (S3L), we demonstrate: (1) Inductive coupling to custom-made saddle coils tuned for various Larmor frequencies. (2) Broadband operation covering the frequency range from ${}^{13}$C to the proton in an 11.74 T NMR scanner (roughly between 125.76 MHz to 500 MHz). This is demonstrated as a proof of concept, however, the S3L structure is able to cover a much broader bandwidth. (3) Up to one order of magnitude NMR signal-to-noise enhancement measured at 500 MHz. (4) Cancellation of the $B_1$-field outside of the focal region by the Lenz effect, leading to precise localisation of the NMR excitation. (5) Manipulation of the $B_1$-field orientation with a 90$^\circ$ phase difference. The stripline Lenz lens rotates $B_1$ by 90$^\circ$ with respect to the driving RF field generated by the saddle coil. This is also useful for applications such as battery structures with driving electrodes, or thin film samples, which then avoids the excitation of unwanted sample regions.

## Results

### Apparatus

**Figure 1:**
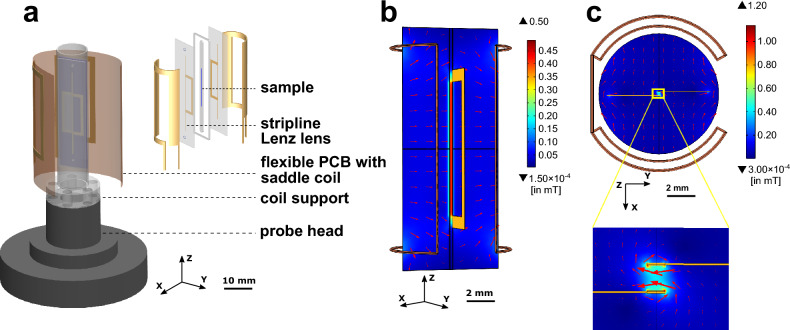
Configuration of a stripline Lenz lens chip on an NMR probe-head, and its magnetic field distribution. (**a**) Illustration of the coil system with a saddle coil and an S3L chip mounted on the probe head. Exploded view of the coils, with the sample region shaded blue. (**b**) B$_{1}$-field distribution on the *xz*-plane via a color map, and an arrow surface plot. (**c**) B$_{1}$-field distribution on the *xy*-plane, and a zoom-in view around the stripline.

Figure 1a shows the measurement probe head including the stripline Lenz lens and the custom-made saddle coil. The S3L structure consists of two striplines running parallel to the main axis of the magnet, each of them having attached an additional rectangular loop on either side of the main axis, respectively. The plane defined by each loop is perpendicular to the $B_1$-field generated by the saddle coil, therefore each loop plays the role of a Lenz lens. It collects the entire RF field incident onto the loop-defined area $B_{1,s}$ and transforms it into a current induced in the metal track of the loop. Consequently, this current generates an RF magnetic field around the metal track. Since the stripline is part of the Lenz lens loop, the narrow sample region defined between two striplines experiences the sum of the RF fields $B_{1,S3L}$ generated in each loop, respectively, as shown in Fig. 1b. Additionally, the Lenz lens structure is used to manipulate the incident RF field by flipping this to $90^{\circ }$, which is presented in the inset of Fig. 1c. The length of each stripline element is extended beyond the insertion point of the loops in order to ensure material continuity and therefore uniform magnetic susceptibility along the *z* axis, i.e., along the static field (see Supplementary Information Sec. §1.3).

For the saddle coil, we have adopted a versatile manufacturing technique based on flexible PCB, which allowed us to design and manufacture multiple transmit coils for various Larmor frequencies. These are used to demonstrate the broadband capabilities of the S3L detector. A rendering of the S3L and of the flexible PCB saddle coils structure is shown in the inset of Fig. 1a, respectively, and full dimensions, as well as a description of the fabrication are given in the *Methods* section.

The S3L is a sandwiched structure of two glass substrates with gold metal tracks for the striplines and the Lenz lens loops defined by a simple process consisting in photolithography, electroplating and etching. An Ordyl dry photoresist layer serves as spacer between the two glass substrates, at the same time defining the sample holder in between the stripline tracks. In the course of the experiment, the signal originating from the sample is detected by the stripline and is inductively coupled to the saddle coil. The entire ensemble S3L and saddle coil is matched to 50 $\Omega$ and tuned to the Larmor frequency of interest using capacitors mounted on the probe head.

### Analytical model of the stripline Lens lens insert

**Figure 2:**
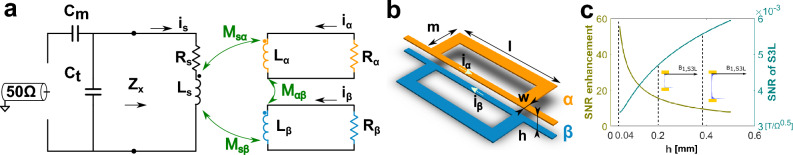
(**a**) Circuit model of the saddle coil, inductively coupled to the S3L insert for SNR enhancement. (**b**) Dimensional parameters of the S3L geometry. (**c**) Calculated SNR enhancement and absolute SNR versus stripline spacing *h* based on the circuit model, and profiles of the $B_1$-field distribution between two striplines (yellow blocks) respectively with $h = 200,~390$ μm.

The signal to noise ratio (SNR) of an NMR detector is a complex figure of merit which is influenced by multiple parameters that take into account the experimental conditions (static field), the sample itself (volume or concentration and nuclei to be detected), and the detector design. An important goal of any NMR experiment is to maximize the SNR. The present section provides an evaluation of the SNR enhancement when using the saddle coil with the S3L insert compared to the situation when only the standard saddle coil is used as a detector for the same sample volume and geometry. This discussion is based on the electrical model of an NMR probe with a Lenz lens stripline pair shown in Fig. 2a. Considering the fact that the total noise is due exclusively to the ohmic resistance of the detector, the SNR enhancement can be written as

1 $$\begin{aligned} \zeta =\frac{B_{1,S3L}/\sqrt{R_x}}{B_{1,s}/\sqrt{R_s}}=\frac{\eta B_{1,s}/\sqrt{\gamma R_s}}{B_{1,s}/\sqrt{R_s}}=\frac{\eta }{\sqrt{\gamma }}, \end{aligned}$$

in which $B_{1,S3L}$ and $B_{1,s}$ are respectively the $B_{1}$-fields generated by the Lenz lens stripline and the saddle coil in the sample region, and $R_{x}$ and $R_{s}$ are the terminal resistances of the saddle coil with and without the S3L insert. The ultimate SNR enhancement is based on the RF field enhancement $\eta$ and the resistance increase $\gamma$. The calculation of Eq. (1) is presented in detail in Supplementary Information Sec. §1.2. This calculation uses the circuit model of the saddle coil, which is inductively coupled with the S3L insert and shown in Fig. 2a. The saddle coil is connected to a tuning and matching network with two capacitors and a 50 $\Omega$ coaxial cable. Upon insertion of the S3L, the voltage measured at the terminals of the saddle coil, $v_{x}$, is modified by the voltages induced due to the coupling between the saddle coil and each of the loops of the S3L ($\alpha$ and $\beta$), as described by the equation below, written for the operating frequency $\omega$

2 $$\begin{aligned} v_{x} = (R_s + j \omega L_s)i_s - v_{\alpha } - v_{\beta }. \end{aligned}$$

The model described in the Supplementary Information also takes into account the symmetry of the problem, which translates into the interchangeability between the indices $\alpha$ and $\beta$, so that the induced voltages are

3 $$\begin{aligned} v_{\alpha } = v_{\beta } = j\omega M_{s \alpha } i_{\alpha } = j\omega M_{s \beta } i_{\beta }, \end{aligned}$$

which determines the terminal impedance of the saddle coil

4 $$\begin{aligned} \begin{aligned} Z_x = R_s + j\omega L_s -2j\omega M_{s \alpha } \frac{i_{\alpha }}{i_s}, \end{aligned} \end{aligned}$$

where $M_{s \alpha /\beta }$ is the mutual inductance between the saddle coil and loop $\alpha /\beta$, and $i_{\alpha /\beta }$ is the current in loop $\alpha /\beta$ ($M_{s \alpha }$=$M_{s \beta }$ and $i_{\alpha }$=$i_{\beta }$), and $R_s$, $L_s$, and $i_s$ are the resistance, inductance and current values corresponding to the saddle coil. Taking into account the actual geometry of the saddle coil, and the S3L loops (see Fig. 2b), as well as the individual $B_1$-field distributions (shown in Fig. 1b,c) for the calculation of mutual inductances, a numerical dependency of the SNR enhancement is derived with respect to the distance between the the two striplines, i.e., between the two planes of the S3L loops.

Figure 2c shows both the SNR enhancement effect as a result of using the S3L insert, and the absolute SNR provided by the S3L insert at 500 MHz, the former calculated by Eq. (1), and the latter from the numerator in Eq. (1) given a unit current. The profiles of the $B_1$-field generated by the striplines (two yellow blocks representing the cross sections) at respectively $h = 200,~390$ μm are also displayed. The results are obtained for a sample volume of 0.4  μL, and for the geometrical parameters of the S3L insert, $w = 200$ μm, $~l = 2$ cm, and $m = 4$ mm, see Fig. 2b.

As expected, there is a significant and rapid increase in the SNR enhancement for small *h* values, i.e., for a small distance between the striplines, due to the $B_{1,S3L}$ being severely restricted between the metal strips. However, the SNR enhancement plot in Fig. 2c does not take into account the proximity effect which becomes important at very low *h* values and which is expected to limit the enhancement effect. As the distance between the striplines increases, the enhancement effect becomes less pronounced, however, it must be noted that it remains around one order of magnitude, even for a relatively large separation of $h=0.5$ mm between the metal strips. The SNR enhancement curve does not depend on the actual amount of sample. The absolute SNR of the S3L insert shows a logarithmic dependency on *h*, i.e., the SNR increase becomes moderate for larger separation between the strips. The curves in Fig. 2c must be always considered in conjunction with the uniformity of the $B_{1,S3L}$-field, and two profile plots of the field strength at $h = 200,~390$ μm are attached as examples. The stripline confinement effect quickly diminishes with increased distance between the strips, the S3L SNR increase being only due to the increase in sample amount. This also means that the $B_1$-field generated by each stripline will be concentrated around the stripline, thus leading to a very non-homogeneous $B_1$-field distribution between the striplines. This optimization is discussed in more detail in the next section.

### Stripline Lenz lens geometry optimization

As for any inductive NMR detector, the performance is described by several figures of merit which are evaluated in this section as a function of the geometrical characteristics of the S3L insert, including the relative SNR, excitation efficiency, $B_1$ homogeneity, and the $B_0$ homogeneity.

The SNR has been thoroughly discussed in the previous section, as well as in the Supplementary Information. The excitation efficiency describes the power transfer from an excitation source to the coil through the terminal, depicted as a 50 $\Omega$ coaxial cable in Fig. 2a. By definition, the excitation efficiency is the frequency of the nutation signal at 1 W excitation power^36^. $B_1$ homogeneity is an important factor for spin manipulation. Poor homogeneity leads to each spin having a different tip angle, i.e., leading to an overall deteriorated sample magnetization which in turn affects SNR. This is commonly evaluated by the ratio of nutation signal intensities at the pulse length of $450^{\circ }$ and $90^{\circ }$, $A_{450^{\circ }}/A_{90^{\circ }}$^20^. $B_0$ homogeneity directly affects the resolution of the NMR spectra and is primarily influenced by any discontinuities in the magnetic susceptibility of the constitutive materials of the NMR probe, especially in the vicinity of the sample. The spectral resolution is evaluated here by the linewidth at 0.11% of full signal height.

The optimization parameter of the S3L structure is the spacing between two striplines *h*. As shown in the previous section and in more detail in the Supplementary Information, *h* regulates the $B_1$-field distribution and the mutual coupling between coils, therefore affects the electrical parameters and the SNR. With a maximal filling factor, the sample volume is calculated to be $(h-2t) \times w \times l$, and it is kept constant throughout optimization for a fair comparison of the SNR. The sample length *l* coincides with the length of the Lenz lens for two reasons. First, any sample outside the Lenz lens suffers from a lower detection sensitivity. Second, there is a susceptibility mismatch at the junctions between the Lenz lens and the stripline (as shown in Supplementary Information Sec. §1.3). As Fig. 1 shows, the stripline extends on both sides of the Lenz lens, and this additional length is considered in our simulation to be filled with susceptibility matching fluid (matched to the acqueous sample).

**Figure 3:**
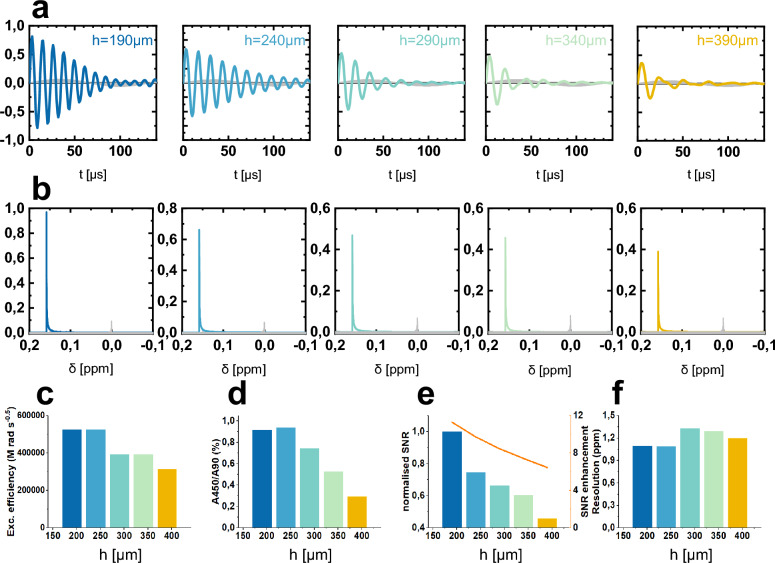
5 different configurations for stripline Lenz lens were simulated in order to determine the optimum spacing between the lobes (*h*). The simulation results were processed to calculate (**a**) nutation signals for different configurations in comparison with the case where no S3L is used (grey signals), (**b**) NMR spectra following a 90$^\circ$-pulse and collected for different configurations together with the case where no S3L is employed (grey spectra), (**c**) excitation efficiency, (**d**) $B_1$ homogeneity, (**e**) relative SNR (left axis) and SNR enhancement (right axis), and (**f**) $B_0$ homogeneity.

The S3L structure optimization was performed on 5 configurations generated by sweeping *h* values between 190 and 390 μm, in steps of 50 μm. The stripline width *w* is adjusted in accordance to each *h* value. A sample volume of 0.4 μL was considered for the signal extraction and was kept constant for all configurations. It is important to note here that, while *h* and *w* were coupled in order to ensure the same sample volume, the effect that each of them has on the NMR results must be discussed independently. The simulation results for the 5 configurations are shown in Fig. 3. The nutation curves and spectra from the same sample geometries without an S3L insert are simulated and added for comparison in Fig. 3a,b.

Figure 3a displays for each configuration the nutation spectrum for the case when the S3L insert is used and for the case when only a dummy insert is considered, i.e., no presence of metal tracks for the stripline or for the Lenz lens structures. The information from Fig. 3a is further processed to generate the bar charts of excitation efficiency in Fig. 3c and $B_1$ homogeneity in Fig. 3d. As expected, the excitation efficiency deteriorated with the increase in stripline separation *h* because of a weaker coupling between two striplines. $B_1$ homogeneity showed a similar trend with increasing separation *h* as the device approaches the situation of two independent wires. Normalised SNR and SNR enhancement shown in Fig. 3e also decreased with the spacing between the striplines. The static field homogeneity is reflected by the spectral resolution, which is crucial in building micro detectors. Figure 3f indicates the best homogeneity when the spacing is around 300 μm.

Figure 3b depicts the simulated NMR spectra and all signal peaks are normalised with respect to the spectrum at $h = 190$ μm. The signal peaks obtained without S3L (grey area) were adjusted to 0 ppm as a reference. Without S3L, the spectra were greatly affected by the low sensitivity, with lower peak magnitudes given bv the same sample amount. In order to compare the spectral resolutions for different configurations, the linewidths at 0.11% full height are compared, the highest resolution around 1.1 ppm being achieved for the first two separation values $h = 190,~240$ μm and being the result of the interplay between *h* and *w*.

Based on these results and as a good compromise among the SNR, spectral resolution, as well as the restrictions imposed by the fabrication (given in Methods section), a stripline spacing of 210 μm and a track width of 200 μm were selected.

### Experiment analysis

**Figure 4:**
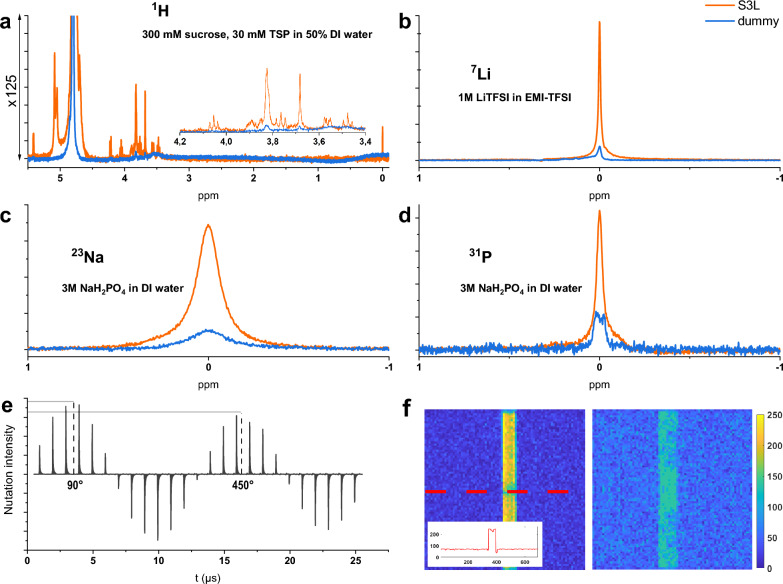
Comparative spectra for different nuclei using a dummy chip, and the broadband S3L add-on. (**a**) Proton ${}^1$H spectra at 500 MHz. The insert shows more detail for the region between 3.4 ppm to 4.2 ppm. (**b**) Lithium ${}^7$Li spectra at 194 MHz. (**c**) Sodium ${}^{23}$Na spectra at 132 MHz. (**d**) Phosphorous ${}^{31}$P spectra at 202 MHz. (**e**) A proton ${}^1$H nutation spectrum obtained using the S3L, corresponding to the orange curve in (**a**). (**f**) MRI of DI water at the center of the stripline with an S3L chip (left) and MRI of DI water with a dummy chip (right). A profile of signal intensity along the red dotted line is addressed.

In order to assess the broadband performance of the S3L insert, four different nuclei were chosen, that together cover a range of Larmor frequencies from 132 to 500 MHz, including ${}^{23}$Na, ${}^7$Li, ${}^{31}$P, and ${}^1$H, with the corresponding 1D spectra presented in Fig. 4. The characterization was performed using the following different samples: 300 mM sucrose, 30 mM TSP in DI water for the proton spectrum, 1 M LiTFSI in EMI-TFSI for ${}^7$Li, and 3 M NaH$_{2}$PO$_{4}$ in DI water for ${}^{23}$Na and ${}^{31}$P. In order to take into account imperfections in the chip fabrication process, such as irregularities in the photoresist-based channel which may have lead to a smaller sample volume, the effective sample volume was corrected to 0.32 μL.

**Figure 5:**
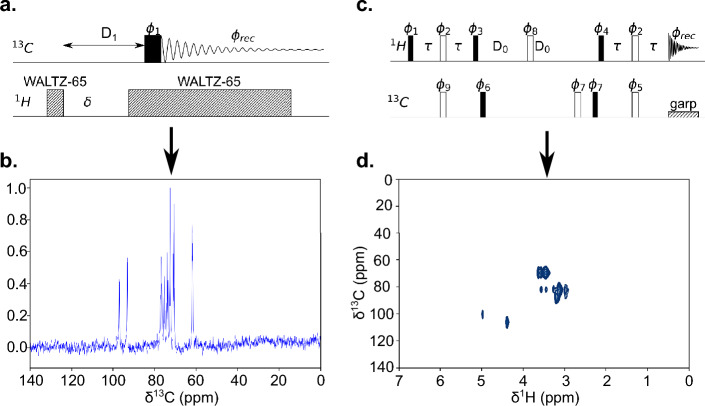
(**a**) Pulse sequence used for the ${}^{13}$C detection with ${}^1$H decoupling. The composite pulse used for decoupling was “Waltz-65”. (**b**) The ${}^{13}$C spectrum obtained from the pulse sequence shown in (**a**). The solution used was 1M 13C-labelled D-glucose in 90:10 D2O/H2O. (**c**) The pulse sequence used for 2D ${}^1$H–${}^{13}$C HSQC. The spectra were recorded without any gradients applied. (**d**) ${}^1$H–${}^{13}$C HSQC spectrum plotted on a 2D scale, with y-axis representing the frequency shift in ${}^{13}$C, and x-axis representing the frequency shift in ${}^{1}$H.

Furthermore, we performed a ${}^1$H–${}^{13}$C HSQC (Heteronuclear Single Quantum Coherence) measurement to present the Lenz lens' promising application in 2D NMR spectroscopy, which is shown as Fig. 5.

The performance of the S3L insert was evaluated by implementing two experimental configurations as shown in Fig. 1. In one configuration, the S3L insert was placed with the plane of the Lenz lens perpendicular to the $B_1$-field of the saddle coil. In the other similarly aligned configuration, a so-called dummy chip that contained only the sample channel, but without a stripline Lenz lens, was used. The following precautions were taken when using the S3L configuration: for a maximum coupling between the saddle coil and the Lenz lens structure, the Lenz lens was fully inserted in the saddle coil and its plane precisely aligned perpendicular to the $B_1$-field of the saddle coil. At the same time, the stripline, together with the sample channel, was arranged rigorously parallel with the static $B_0$-field.

#### 1D NMR with multiple isotopes and MRI experiments

Figure 4 summarizes the NMR spectra with four different isotopes, and nutation signals recorded from ${}^1$H. For a clearer visual comparison, the spectra with the same isotope were scaled to have the same noise level, i.e., the same baseline height. The peak corresponding to TSP was set at 0 ppm in the proton spectrum, while the peaks corresponding to ${}^{23}$Na, ${}^7$Li, and ${}^{31}$P in the respective spectra were set to 0 ppm. The key experimental parameters are summarised in Table 1 for a quantitative comparative evaluation.

As presented in Fig. 4a, a zoomed view along with a full view shows the ${}^1$H signal enhancement with the S3L chip inductively coupled to the saddle coil. From these two spectra obtained with 10 W excitation power and a receiver gain of 32, the respective SNR of the singlet water peak at 4.8 ppm was calculated. The single scan SNR enhancement between a perpendicular S3L chip and dummy chip, i.e., the orange and blue curves, was ${7030}/{628} = 11$. With the S3L chip facing the $B_1$-field, the pulse length was cut down from 27 to 3 ms showing a 9-fold field enhancement. In addition, the linewidth of the peak with a perpendicular S3L chip was narrowed from 6 Hz down to 3 Hz, which reflects an improved field homogeneity. The shim condition was calibrated separately for each setup. Supplementary Information Table S1 presents a comparison of the frequency-domain normalised limit of detection (nLOD) at 14.1 T between the S3L device and other NMR micro-detectors previously reported in the literature. The S3L add-on featured an equivalent nLOD value calculated at 600 MHz of 18 nmol/ $\sqrt{s}$, which was slightly lower compared to the NMR micro-detectors, keeping in mind however that all other detectors are active (tuned resonator) devices, whereas the S3L insert operated in an inductively passive mode. The S3L thus compares favourably in terms of spectral resolution (around 6 *ppb* in the benchmarking proton measurement) and $B_1$-field homogeneity^4,7,10,11,14,37^.

Figure 4e presents the nutation spectrum acquired using a water sample, in order to determine the 90-degree pulse length, and to assess the field homogeneity by calculating the ratio ${A_{450^{\circ }}}/{A_{90^{\circ }}}$. This revealed that the 90-degree pulse length when using the S3L insert was half compared to when using a dummy insert (3.1 ms versus 14 ms), which implied a 5-fold excitation efficiency for a good $B_1$-field homogeneity of 85.8%.

${}^7$Li experiments displayed a single peak from the LiTFSI sample, as displayed in Fig. 4b. Switching from the dummy chip to the S3L chip, the SNR enhancement was around 8.4, reaching an SNR of 402 with 512 averages, due to the lower NMR sensitivity and an nLOD of 82 nmol/ $\sqrt{s}$. At the same time, the linewidth dropped from 4.6 to 2.4 Hz. From the nutation spectrum (not displayed here), ${A_{450^{\circ }}}/{A_{90^{\circ }}}$ was around 81%.

${}^{23}$Na and ${}^{31}$P spectra (Fig. 4c,d respectively) were obtained with 3 M NaH$_{2}$PO$_{4}$ solution in DI water. The relatively high concentration of this sample was necessary due to its even lower molar receptivity than ${}^7$Li. As shown in Table 1, the ${}^{23}$Na signal had an enhancement of around 6.6 and the ${}^{31}$P signal had an SNR enhancement of 6.8.

MRI experimental results with two different chips are presented in Fig. 4f, in which distilled water was used as the sample. Each image has $80 \times 80$ pixels with a pixel resolution of 25 μm, and is measured from a slice thickness of 200 μm. The pixel value is scaled to a range of 0–250. The repetition time and the excitation power were adjusted towards a best SNR, respectively 1 s and 0.18 mW for the S3L chip, and 1 s and 20 mW for the dummy chip. It is noted that the dummy chip result was acquired with 16 averages, which brought a 4-fold SNR enhancement. As shown in the images, some spots appear to have weaker signals due to air bubbles formed in the water sample after continuing the experiments. A profile of the signal amplitude across the sample region is also attached, which reflects directly the signal and noise level collected with one pixel. A sharp boundary of the signal area is observed due to the metal strip constrains.

For the SNR calculation, regions of interest (ROIs) are defined on no-sample regions as "noise", and on sample regions as "signal". SNR is defined as the ratio between the mean value in the sample region, and the root mean square value in the noise region. For a better accuracy, four square ROIs are measured in four corners of the image around the stripline. An MRI SNR of average a is about 11.6 for the S3L chip, and around 1.38 for the dummy chip, which shows an enhancement of 8.4, which has a good correspondence with the NMR result as expected. This slightly smaller enhancement value compared to the NMR proton experiment is mainly caused by bubbles in the fluidic channel.

**Table 1:** NMR broadband reception at frequencies of different nuclei in an 11.74 T field.

Isotopes	${}^1$H		${}^{31}$P		${}^{7}$Li		${}^{23}$Na	
Larmor frequency (MHz)	500		202		194		132	
Relative molar receptivity	1.00		0.07		0.29		0.09	

Configuration	S3L	Dummy	S3L	Dummy	S3L	Dummy	S3L	Dummy
Power (watt)	10	10	10	10	10	10	10	10
Pulse length (ms)	3	27	15	15	30	30	10	10
Number of scans	1	1	128	128	512	512	256	256
Relaxation delay (s)	10	10	5	5	1	1	5	5
Linewidth (Hz)	3.1	6.2	6.8	14.6	4.6	6.8	19.6	34
Measured SNR	7030	628	68	10	402	48	79	12
nLOD (spins $\sqrt{s}$)	18	204	914	6237	56	465	1038	7171
SNR enhancement	11.2		6.8		8.4		6.6	

#### Double-channel NMR pulse sequences on the S3L

NMR is powerful tool to predict the structure of a molecule. Though most structural information can be extracted from single RF channel experiments, yet a double channel RF experiment may help to reveal obscured information. The two most used pulse sequences are heteronuclear, of which one class is referred to as decoupled sequences, the other as coherence spectroscopy experiments. Therefore, it is important for a suitable resonator, in the present case the S3L, to be capable of also handling heteronuclear experiments.

For the purpose of demonstrating the S3L capabilities, a double-channel driving saddle coil was designed, where the resonances for the nuclei ${}^1$H and ${}^{13}$C were achieved using trap circuits as explained in^38^. This choice is convenient, since depending on the target nuclei, only the double channel trap circuits need to be re-adjusted accordingly. The design of the S3L reported in this paper is therefore independent of these resonant frequencies.

Two types of pulse sequence were applied, ${}^1$H-decoupled ${}^{13}$C detection, and ${}^1$H–${}^{13}$C HSQC. The graphical representation of the pulse sequences is shown in Fig. 5a,c.

The spectra shown in Fig. 5b,d were acquired using an S3L, where the sample used was 1 M ${}^{13}$C labeled D-glucose in 90:10 D_2_O/H_2_O. The pulse parameters for the experiments are summarized in Tables 2 and 3. Without the the S3L, spectra of the measurements described could not be acquired due to low signal levels.

**Table 2:** Pulse parameters for ${}^{13}$C NMR with ${}^1$H decoupling.

Parameters	Values
RF power	25 W
90$^\circ$ flip angle pulse length	10 μs
Number of scans	128
Decoupling power	0.7 W
Decoupling length	90 μs
Recycle delay D$_{1}$	1 s
$\delta = D_{1} - 100$ms $\delta$	900 ms

**Table 3:** Pulse parameter for ${}^1$H–${}^{13}$C HSQC.

Parameters	Values
${}^1$H RF power	20 W
${}^1$H 90$^\circ$ flip angle pulse length	6 μs
Number of data points in FID	4096
${}^{13}$C RF power	25 W
${}^{13}$C 90$^\circ$ flip angle pulse length	10 μs
Number of data points in FID	512
Decoupling power	0.5 W
Decoupling length	70 μs
Number of scans	8
J-coupling constant (J)	145 Hz
$\tau =(4J)^{-1}$	1.72 ms
$\text {Increment delay D}_{0}$	3 μs

## Discussion

Micro-coil NMR has long been a pursued direction in this field, to obtain a better sensitivity, along with the practical demand for a reduced necessary sample volume. Though various coil geometries are already well studied for different application scenarios, it is not always easy to design and produce a to-the-point micro-coil for constantly emerging situations in one lab. Considering the high cost of implementing an NMR device, and the difficulty in daily maintenance, it would be most helpful if the device can be simply handled, is compatible with different NMR coils, and even better, work in different frequency ranges, i.e., applicable to different nuclei on an identical magnet, or on different magnets.

In this work, we have shown that a stripline Lenz lens add-on can be a brand new solution to the dilemma above. While the stripline is a more mature design as NMR coil, the Lenz lens is still less studied in research and their combination gives a simple yet effective geometry capable of focusing the $B_1$-field generated by an external coil into the stripline space, and re-orienting the field flux by rotating it by 90$^\circ$. As the calculation shows, the field strength can be enhanced up to 20 times while the SNR enhancement is up to 11 depending on the spacing between the striplines. For NMR coils of different sizes, the dimension of the add-on is easily adjustable so that a maximal coupling is ensured, and this performance can be replicated. Yet, the possible imperfections from the fabrication process should be always considered for extreme sizes, e.g., the minimum etchable sample channel width.

Using an S3L add-on, we have observed a good agreement between the theoretical analysis and experiment impovement in the RF field and sensitivity enhancement. With around 0.32 μL sample, we realized an SNR enhancement of one order of magnitude on proton measurements. Along with the SNR improvement, the S3L add-on greatly reduced the 90$^\circ$ pulse length, verified by nutation measurements. For the other three isotopes with lower receptivities, i.e., ${}^{7}$Li, ${}^{31}$P, and ${}^{23}$Na, the add-on device brought a relatively lower SNR improvement of around 7–8. The main reason is that we simplified the operation and applied a same moderate pulse length, instead of respective 90$^\circ$ pulses, in order to check the direct change with the S3L chip. Another general cause is the lower NMR performance of the customized saddle coil, which only has a single loop, compared to commercial coils with two loops and hence a higher RF excitation efficiency. Additionally, the spectral resolution improved by a factor of four in respective experiments of the four isotopes.

These findings have a number of implications. At the expense of sensitive sample volume, SNR is greatly enhanced, which can be utilized to selectively perform NMR measurements on small regions of interest of technical films, or cylinder-like samples, e.g., biological biopsy tissue plugs. Furthermore, it has potential as a broadband implant for in vivo experiments.

With a response ranging from 125.76 to 500 MHz at 11.7 T, the broadband capability of the Lenz lens stripline was qualified for most nuclei of interest. The excitation coil possesses a tunable resonance frequency, which can be disturbed by the inserted stripline chip. Depending on the adjustment range of the capacitors on the probe, it could possibly happen that the tuning and matching condition is not perfectly reached when an S3L add-on is loaded. In such a case, the coil reflects more power and has a lower quality factor (Q), leading to a longer excitation pulse length for a given power, and hence worse sensitivity. In our design, the total terminal resistance was however far smaller than 50 $\Omega$, so that it could be rematched. the terminal inductance change remained within 5%, thus any detuning could be easily compensated on the probe.

The Lenz lens redirected the $B_1$-field of the saddle coil by 90$^\circ$ into the sample space, at the same time eliminating the $B_1$-field everywhere else based on the Lenz effect. This aspect has great potential for future studies. As one typical example, a battery sample is covered by metal electrodes and suffers from low filling ratio inside a normal NMR resonator. The flipped field generated by a Lenz lens stripline can circumvent the electrical shielding, and precisely excite the battery material. Thus this add-on is highly adoptable for non-invasive in-situ battery studies.

## Methods

### FEM simulation in COMSOL MultiPhysics

Through the process of evaluating the RF field by the S3L chip, and optimising the detector geometry, COMSOL MultiPhysics 5.6 (COMSOL AB, Sweden) was employed together with the RF module (Electromagnetic Waves, Frequency Domain).

For the geometry model, the reference was established with a 10 mm saddle coil without an S3L inserted, in comparison with the case with the chip placed at the center of the saddle coil. The sample channel was fixed to be 0.6 μL, with a constant length of 1.4 cm, whereas the cross section shape depended on the variable *h*. The material of the coil structure adopted COMSOL's built-in electroplated gold, and the open space was applied as air. The sample material was set to be liquid water, which was specified with an electrical conductivity of 0.005 S m$^{-1}$ and a relative permittivity of 80.2. The coil system was located inside a spherical space with a diameter of 6 cm, which was assigned as a perfect electric conductor at the boundary for characterisation of the magnetic field inside. A free tetrahedral mesh was applied to the whole domain, where a maximum element size of 250 μm was employed on the coil structure and sample region.

We swept the stripline spacing *h* at 500 MHz for water measurement. As the excitation source, a lumped port with 1 A terminal current was connected to the saddle coil model. The relative tolerance of the RF simulation was 1e-4.

The results were processed in MATLAB R2018a (MathWorks Inc., USA) for Fig. 3. The processing manuscript is available upon request.

### Saddle coil fabrication with flexible printing circuit board (PCB)

**Figure 6:**
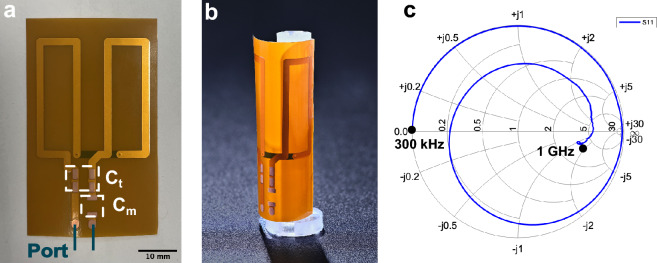
(**a**) Low frequency homonuclear saddle coil printed on a flexible PCB, which had buried coil wires and an exposed solder pad for additional capacitors. For high frequency homonuclear and heteronuclear experiments, a suitable double resonant trap was added and the saddle coil topology adapted to a parallel design^38^. (**b**) PCB partially wound coil on a 3D-printed support frame. (**c**) Smith chart of a bare saddle coil from 300 kHz to 1 GHz without tuning and matching network, measured on an E5071C network analyzer (Agilent Technologies, California, United States).

For 1D proton measurements at 500 MHz, a Bruker 10 mm saddle coil was available, while for other isotopes with lower frequencies we fabricated coils in-house^39,40^. As shown in Fig. 6, the customized saddle coil was made through wrapping a flexible PCB sheet on a 3D-printed tube. In comparison to other tested fabrication methods, e.g., winding copper wire on a plastic tube, the flexible PCB was easier to maintain a perfect saddle configuration with good reproducibility, and thus a higher field homogeneity was ensured.

The three-layer flexible PCB substrate was 217 μm thick (Multi Leiterplatten GmbH, Germany). The conductor track on the board was 35 μm thick and 2 mm wide, and two conductor layers were utilized in our S3L layout. The insulation polyimide layer in-between was 25 μm. The transparent supporter tube was 3D-printed from ABS filament on an Ultimaker 2+ (Ultimaker B.V., Netherlands) with a 4 mm nozzle, which had a base compatible with the pins on the NMR probe head. The rolled-up saddle coil had a height of 20 mm, a diameter of 10 mm and an aperture angle of 120$^\circ$ based on a previous study^41,42^. The vertical position of the coil met the homogeneous space of the static field.

The self resonance frequency of the custom coil was around 460 MHz, which could be further tuned for most nuclei, but was not appropriate for ${}^{19}$F at 470 MHz. On the other hand, the limited range of capacitor values caused problem throughout the tuning and matching adjustment. Additionally, if one strived to realise a lower resonance frequency than 100 MHz, high-value capacitors posed bigger tolerances, thus increasing the uncertainty throughout the tuning and matching adjustment. The capacitances $C_t$ and $C_m$ required to tune the ${}^7$Li-detection coil to 194 MHz were respectively 5.6 pF and 7.5 pF. For ${}^{23}$Na the saddle coil required 15 pF and 15 pF, and for ${}^{31}$P at 202 MHz, the saddle coil required 5.1 pF and 7.5 pF.

### Microfabrication of S3L chip

**Figure 7:**
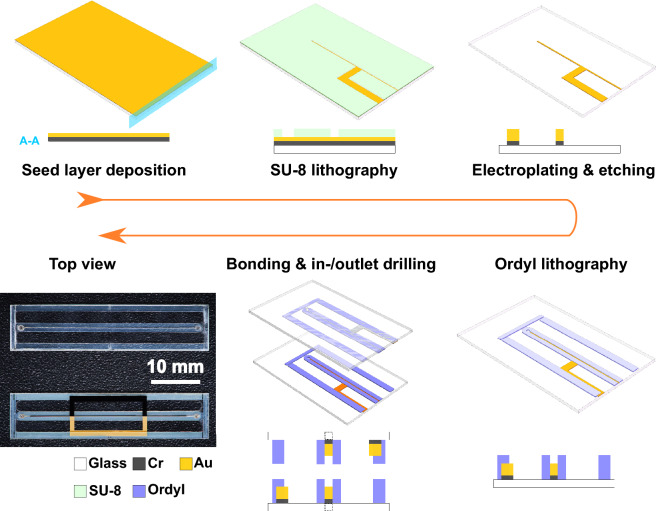
Fabrication process of S3L chip with both 3D model and 2D cross-section, as well as a top view of the fabricated S3L chip alongside with a dummy chip.

Adopting the optimised geometry from the section above, *Stripline Lenz lens geometry optimization*, the S3L chip was fabricated in our in-house cleanroom following the steps shown in Fig. 7. Due to the geometric symmetry, only half of the chip is presented in the figure and it is noted the height dimension in the plot is not to scale for a better visualisation of the layer stack.

- *Seed layer deposition* The stripline Lenz lens chip is a sandwiched structure which consists of two 200 μm 4” D263 Teco gold-coated glass wafers and an around 200 μm thick Ordyl layer (a dry film photoresist) in between. The glass wafer was first cleaned with a typical routine in the cleanroom by Acetone and Isopropanol and then exposed to Oxygen plasma for 5 min. The wafer was later coated with chromium/gold seed layers (20 nm and 60 nm) for following growth of gold structures.
- *SU-8 lithography* Standard SU-8 lithography process was applied on the top side for patterning the gold Lenz lens and stripline. The thickness of the photoresistwas around 20 μm.
- *Electroplating & etching* With the seed layer connectd to a DC source, around 20 μm thick gold was electroplated in the SU-8 mould. After the SU-8 was stripped off, the seed layer area, which was not covered by the stripline Lenz lens, was also etched away.
- *Ordyl lithography* On the top of the gold structure, an Ordyl layer of around 110 μm (90 μm + 20 μm) was directly laminated using an office laminator. The microfluidic channel and the supporting sidewall of the chip were patterned using a lithography process, and developed in Ordyl SY developer for 20 min assisted by an ultrasound bath.
- *Bonding & drilling the inlet and outlet* Ordy loses its stickiness and gets dried easily in the air. After development and necessary cleaning of the surface, the wafer was exposed to oxygen plasma for a very short time for a better bonding quality. Taking advantage of the geometry symmetry, we flipped one out of the two identical wafers and covered it with the other. The assembly was aligned under the microscope aided by the align markers and fixed with thermal tapes, and then brought to bonding employing a compression-bonding machine (EVG510 EV Group) under 5 kN force at 95 $^\circ$C for 4 h. The inlets and outlets were cut with a nanosecond laser (PIRANHA ACSYS) into the top layer, with a hole diameter of around 260 μm. The entire wafer assembly was then diced into individual chips using the laser, and one batch with two 4” glass wafers was able to produce 12 identical assembled chips.

The final S3L device is shown from the top in Fig. 7, next to a fabricated dummy chip. For the dummy chip, the steps from seed layer to gold electrodeposition are excluded. Due to the flexibility of the Ordyl layer, the thickness difference of the whole chip introduced by the extra gold tracks could be neglected after the bonding process.

Ordyl etching quality is a major concern, which is related to the sample capacity and the bonding stability. Etching performance depends mainly on the structure geometry, substrate material, exposure amount, and etching time. An etched channel with a high aspect ratio normally caused a width difference between the top and the bottom surfaces of the resist layer. It also happened that the etching solution could not remove the substance completely from the exposed Ordyl area, and then created a clogged channel. Thus, the flip-and-assembly method, i.e., two wafer pieces each with half an aspect ratio, was beneficial to the fabrication quality and also simplified the process despite the extra effort in the layout. As we cut the chip and characterised the cross section under a microscope, the sample channel in an S3L chip was around to be around 160 μm wide and had a vertical sidewall. However, a dummy chip had an inverted trapezoid cross section, with a short base of around 100 μm on the glass and a long base of 160 μm. Hence, the sample volume in the dummy chip was around 15% less than in an S3L chip.

### NMR and MRI characterisation

The NMR/MRI experiments were conducted on an 11.74 T Avance III Bruker NMR system (Bruker BioSpin, Rheinstetten, Germany) with a Micro5 micro-imaging probe and a gradient sleeve. For sample loading, due to the rough surface and edge of the laser-cut holes and Ordyl sidewalls, the capillary effect was not sufficient for sample loading despite a micron-sized channel. Thus, we used a syringe with polymer tubing at the tip to inject the sample solution into the channel. The inlet and outlet were sealed with a small piece of tape. The detector assembly of the loaded chip and saddle coil was then connected onto the probe and aligned with the assistance of a PMMA supporter so that the chip was vertically parallel to the static $B_0$-field and horizontally perpendicular to the RF $B_1$-field. In the mannual operation, an alignment error from the perpendicular direction was smaller than 11$^\circ$, which was caused by the thickness of the supporter stick.

For NMR measurement operation, and spectral processing, the software TopSpin 3.5pl2 was applied. ${}^1$H signals were routed through a narrow-band pre-amplifier (${}^1$H LNA MODULE 500, Bruker BioSpin, Rheinstetten, Germany) with a 1 dB noise figure, while X-nuclei signals were routed through a broadband pre-amplifier (XBB19F 2HS MODULE 500, Bruker BioSpin, Rheinstetten, Germany) with a 2 dB noise figure. The temperature was measured to be 30 $^\circ$C by the system. Main NMR acquisition data for all isotopes are presented in Table 1.

For MRI measurements, ParaVision 6.0.1 was used. A flash sequence was applied with modified settings for the illustrated MRI results in Fig. 4f. The repetition time was set to 1 s, the image size was 2 mm $\times$ 2 mm with a resolution of 0.025 mm, the measured slice thickness was 0.2 mm, and the bandwidth was 20 MHz. The images were post-processed in MATLAB R2018a (MathWorks Inc., USA).

### Supplementary Information

Supplementary Information.

## Data Availability

The data and material that support the findings of this study are available from the corresponding author upon request.
