# Supplementary material for: Broadband stripline Lenz lens achieves 11 × NMR signal enhancement
Source: Sci Rep. 2024 Jan 18;14:1645. doi: 10.1038/s41598-023-50616-0 (PMC10796323; doi:10.1038/s41598-023-50616-0)
Supplement: Supplementary file 1 — Supplementary Information. [file 41598_2023_50616_MOESM1_ESM.pdf]

§1 Supplementary Information

§1.1 A summary diagram of NMR micro coils

Below we summarized different stripline coil researches together with the S3L add-on chip presented in this paper, regarding sample selection, working frequency and experimental performances, i.e., nLOD, spectral resolution (FWHM) and RF homogeneity in 1 H NMR experiment. It is noted that the nLOD is normalised to 600 MHz and the RF field homogeneity is calculated from $A_{450^\circ}/A_{90^\circ}$ if not specified.

Table S1: A summary of micro NMR detector studies.

Source	nLOD $nmol/\sqrt{s}$	FWHM Hz	Sample	Volume nL	f MHz	RF homogeneity
Bentum et al.[13]	1.2	50	ethanol	12	600	62%
Bart et al.[1]	0.47	0.7	600 nmol sucrose in D_2O	600	600	76%
Ryan et al.[10]	0.95	4.5	5% v/v ethanol in D_2O	1200	600	35%
Finch et al.[5]	1.57	1.73	150 mM sodium acetate in H_2O	2000	300	50–95%
Chen et al.[4]	0.73–1.21	1.2	0.2 mM sucrose in H_2O	0.63	500	ca. 75%
Sorte et al.[12]	NA	2	1 M $LiPF_6$	2000	400	67%
Sharma et al.[11]	1.4	3.35	130 µM sodium acetate in H_2O	2000	500	92%*
Krojanski et al.[7]	0.39	3.6	0.215 M sucrose in D_2O	10.6	500	78–97%
Oosthoek et al.[8]	0.5–0.7	3	0.5 mM ethyl crotonate	150	600	NA
**S3L add-on**	**18**	**3.1**	300 mM **sucrose in DI H_2O**	**320**	**500**	**86%**

*The ratio is calculated at 810°.

§1.2 Electrical model of the resonator

In this section, we present the full derivation and calculation of the equations shown in Sec. **Analytical model of the stripline Lens lens insert**. As shown in Fig. 2 (a), we have the saddle coil and two identical Lenz lens loops modeled as RL circuits respectively. Mutual inductance exists between any two of the three coils. H and D are respectively the height and the diameter of the saddle coil, conventionally 20 mm and 10 mm for a wide range of NMR magnets. To best fit in the saddle coil with a maximum filling factor, we assume the side lengths of the Lenz lens loop to be $l \times m = 16mm \times 4mm$, the width of the middle stripline w to be 200 µm and the rest segments to be 6 times wider, i.e., 1200 µm based on typical stripline design rule[2], which increases the current density inside the central stripline by 6-fold. 20 µm is adopted for the track thickness t considering the skin depth effect at a working frequency at 500 MHz and the fabrication constraints.

Based on previous theoretical study of Lenz lens device[6], the voltage seen at the terminal of the "loaded" saddle coil v_x, i.e., when the S3L is inserted as shown in Fig. S1, is given by the difference between the voltage of the "unloaded" saddle coil, i.e., without the S3L, and the induced voltages due to the coupling between the saddle coil and the each of the loops of the S3L, alternatively loop α and loop β

$$v_x = (R_s + j\omega L_s)i - v_\alpha - v_\beta, \tag{S1}$$

in which R_s and L_s are the resistance and inductance of the saddle coil, i is the current inside the saddle coil, ω is the working frequency.

By definition, the induced voltages depend on the mutual inductance between the saddle coil and loops α and β (Eq. S2). Due to the symmetry reasons, electrical parameters denoted with α and β have the same values, i.e., $v_\alpha = v_\beta$, and also $R(L)_\alpha = R(L)_\beta, M_{s\alpha} = M_{s\beta}, M_{\alpha\beta} = M_{\beta\alpha}$ and $I_\alpha = I_\beta$ appearing in following equations

$$v_\alpha = v_\beta = j\omega M_{s\alpha} I_\alpha. \tag{S2}$$

$M_{s\alpha}$ represents the mutual inductance between the saddle coil and either Lenz lens loop, and I_α is the current in one Lenz lens loop. Thus, the impedance seen at the terminals of the "loaded" saddle coil is given by the ratio between the voltage at the terminals of and the current in the loaded saddle coil in Eq. S3[6]

$$Z_x = \frac{v_x}{I_s} = R_s + j\omega L_s - 2j\omega M_{s\alpha}\frac{I_\alpha}{I_s}. \tag{S3}$$

Here, $v_{s\alpha}$ is the voltage induced in loop α by the saddle coil. This can be written as Eq. S4 taking into account also the mutual coupling between the two loops of the S3L

$$v_{s\alpha} = I_\alpha(R_\alpha + j\omega L_\alpha) - v_{\alpha\beta}, \tag{S4}$$

where R_α and L_α are the resistance and inductance of one Lenz lens loop, and $v_{\alpha\beta}$ is the voltage induced in one loop α by the β. The induced voltages have also been expressed according to the definition as a function of mutual inductance in Eq. S5

$$v_{s\alpha} = j\omega M_{s\alpha} I_s, v_{\alpha\beta} = j\omega M_{\alpha\beta} I_\alpha, \tag{S5}$$

where $M_{\alpha\beta}$ is the mutual inductance between two Lenz lens loops. With the current in loop α being expressed as Eq. S6 according to Eq. S4, we obtain the ratio of the two currents after some algebraic calculation as Eq. S7, which is one of the ratios used to calculate the SNR enhancement in Fig. 2 (c)

$$I_\alpha = \frac{v_{s\alpha} + v_{\alpha\beta}}{R_\alpha + jwL_\alpha} = \frac{j\omega(M_{s\alpha} I_s + M_{\alpha\beta} I_\alpha)}{R_\alpha + j\omega L_\alpha}, \tag{S6}$$

$$\frac{I_\alpha}{I_s} = \frac{j\omega M_{s\alpha}}{R_\alpha + j\omega(L_\alpha - M_{\alpha\beta})}. \tag{S7}$$

We replace these current ratios in the expression of impedance from Eq. S3, and Z_x now is described by Eq. S8

$$Z_x = R_s + j\omega L_s + \frac{2\omega^2 M_{s\alpha}^2}{R_\alpha + j\omega(L_\alpha - M_{\alpha\beta})} \triangleq R_s + jwL_x. \tag{S8}$$

The real part can be isolated as in Eq. S9

$$R_x = R_s + \frac{2\omega^2 M_{s\alpha}^2 R_\alpha}{R_\alpha^2 + \omega^2(L_\alpha - M_{\alpha\beta})^2}. \tag{S9}$$

We further express the ratio of the two resistances, where each value on the right-hand side of the equation is being calculated individually starting from the actual dimensions of the saddle coil and loop, respectively

$$\frac{R_x}{R_s} = 1 + \frac{2\omega^2 M_{s\alpha}^2 R_\alpha}{R_s\left(R_\alpha^2 + \omega^2(L_\alpha - M_{\alpha\beta})^2\right)} \triangleq \gamma. \tag{S10}$$

This is the second ratio that makes up the SNR enhancement. According to the reference[3], the B_1-field at the geometric center of a saddle coil is given by Eq. S11, which is the sum total of the magnetic fields by four arcs and four strips. In addition, it can be seen as homogeneous considering the ultra small sample volume compared to the saddle coil space

$$\begin{aligned} B_{1,s} &= 4(B_{1,arc} + B_{1,strip}) \\ &= \frac{\sqrt{3}\mu_0 D I_s H}{4\pi}\left(\frac{1}{(D^2/4+H^2/4)^{3/2}} + \frac{1}{D^2/4(D^2/4+H^2/4)^{1/2}}\right) \\ &= \frac{\sqrt{3}\mu_0 I_s H\left(D^2/2+H^2/4\right)}{\pi D\left(D^2/4+H^2/4\right)^{3/2}}, \end{aligned} \tag{S11}$$

in which $B_{1,arc}$ and $B_{1,strip}$ are field components from one arc and one strip in the saddle coil. μ_0 is the magnetic permeability in air, equal to $4\pi \times 10^{-7}\mathrm{H/m}$.

As shown in Fig. 1 (b), the RF field in S3L, $B_{1,S3L}$, is confined by two parallel stripline segments and can be calculated according to Biot Savart's Law from two current-carrying conductors. The local field strength at distance x from one stripline surface is calculated and further integrated between two striplines for the average field strength between two striplines

$$
\begin{aligned}
B'_{1,S3L} &= \frac{I_\alpha \mu_0}{2\pi}\left(\frac{1}{x}+\frac{1}{h-x}\right), \\
B_{1,S3L} &= \frac{1}{h-2t}\int_t^{h-t} B'_{1,S3L}\mathrm{d}x \\
&= \frac{I_\alpha \mu_0}{2\pi(h-2t)}\ln\left(\frac{h-t}{t}\right),
\end{aligned}
\tag{S12}
$$

in which t is the thickness of the track. The integration range starting from t is for the valid sample region and it avoids the calculation error by applying zero in logarithmic equation. A combination of Eq. S11 and Eq. S12 generates the ratio $\eta = B_{1,S3L}/B_{1,s}$, which is plotted in Fig. S1 (a).

Before proceeding the numerical result of R_s/R_x and I_α/I_s, we need to calculate the unknown parameters in the equations, i.e., R_s, R_α, L_α and $M_{s\alpha}$, $M_{\alpha\beta}$. The AC resistance and inductance of the Lenz lens loop, R_α and L_α can be determined by following empirical equations for the self-resistance and inductance of a rectangular strip conductor[9]

$$
\begin{aligned}
R_\alpha &= \frac{l}{2(a+t)\delta\sigma}, \\
L_\alpha &= 2l\left(\ln\frac{2l}{a+t}+0.5+0.2235\frac{(a+t)}{l}\right),
\end{aligned}
\tag{S13}
$$

where δ is the skin effect depth, σ is the conductivity. As the saddle coil is a standard NMR detector, the calculation of its impedance values are not discussed here. By applying dimension values above to the Eq. S13, R_s, R_α, L_α are respectively 0.546 Ω, 0.265 Ω and 35.5 nH at 500 MHz for proton measurement.

The parameters still missing are the mutual inductance between one single loop and the saddle coil $M_{s\alpha}$, and the mutual inductance between two loops $M_{\alpha\beta}$. The magnetic flux differential is proportional to the field strength differential and a constant area of the receiving loop as Eq. S14

$$\mathrm{d}\Phi_{s\alpha} = \mathrm{d}AB_s. \tag{S14}$$

The relationship between the magnetic flux received by a single Lenz lens loop and the mutual inductance is defined as Eq. S15

$$\frac{\mathrm{d}\Phi_{s\alpha}}{\mathrm{d}t} = \frac{\mathrm{d}I_s}{\mathrm{d}t}M_{s\alpha}. \tag{S15}$$

In the equations above, $\Phi_{s\alpha}$ is the saddle-generated magnetic flux onto the loop and A is the area of the secondary coil perpendicular to the magnetic flux. $A = l \times m \approx \frac{1}{2}DH$ when the Lenz lenses take up a maximal cross section area inside the saddle coil.

By combining Eq. S11, S14 and S15, we obtain the expression of $M_{s\alpha}$ and further exact values with the given dimensions of the saddle coil and the Lenz lens loops

$$M_{s\alpha} = \frac{B_{1,s}}{I_s}A = \frac{\sqrt{3}\mu_0 H^2\left(D^2/2+H^2/4\right)}{\left(2\pi D^2/4+H^2/4\right)^{3/2}} \approx 1.49\times 10^{-8}. \tag{S16}$$

The magnetic flux from one loop onto the other is equal to the flux difference by two long-side strips in the loop carrying opposite-direction currents, denoted as Eq. S17, while two short-side strips are not affecting the coupling. The differential magnetic flux by an l long conductor from the loop can be calculated as Eq. S18b according to Biot Savart's Law and is integrated in respective distance ranges for Φ_1 and Φ_2

$$\Phi_{\alpha\beta} = \Phi_1 - \Phi_2. \tag{S17}$$

The flux terms are described by

$$\mathrm{d}\Phi = \frac{\mu_0 I_\alpha}{2\pi r} l \mathrm{d}x, \tag{S18a}$$

$$\Phi_1 = \int_0^m \frac{\mu_0 I_\alpha l}{2\pi\sqrt{x^2+h^2}} x\mathrm{d}x, \tag{S18b}$$

$$\Phi_2 = \int_m^{2m} \frac{\mu_0 I_\alpha l}{2\pi\sqrt{x^2+h^2}} x\mathrm{d}x, \tag{S18c}$$

in which r is the distance from the loop to the strip. In a similar way as Eq. S14 and S15, $M_{\alpha\beta}$ can be calculated from Eq. S18 as following:

$$\begin{aligned} \frac{\mathrm{d}\Phi_{\alpha\beta}}{\mathrm{d}t} &= \frac{\mathrm{d}I_\alpha}{\mathrm{d}t} M_{\alpha\beta}, \\ M_{\alpha\beta} &= \frac{\mu_0 l}{4\pi}\left(2\ln(m^2+h^2) - \ln(h^2) - \ln(4m^2+h^2)\right). \end{aligned} \tag{S19}$$

Since two mutual inductances are clear now, we get back to the Eq. S7 and Eq. S10 and then, R_s/R_x over the change of h is shown in Fig. S1 (b). Now we combine two elements together and obtain the SNR enhancement at 500 MHz, as shown in Fig. 1 (b) in the main text. The results show that a dominant SNR enhancement comes from the RF field enhancement due to the inserted S3L chip. However, a higher resistance at the saddle coil, which in fact degrades the SNR slightly.

§1.3 B$_0$ field distribution analysis along sample channel

In this section, we explore the influence of material interfaces on B_0-field distribution along the sample channel through COMSOL simulation. The goal is to check the active sample volume inside a homogeneous static field.

A static magnetic field of 11.7 T is applied in the space and the field strength at the center of the sample channel is plotted in Fig. S2. The full channel length within the Lenz lens is utilized as the active sample region while at two ends of the sample channel, sample volume is further extended with a constant length of 0.01 m designed as a susceptibility-matching plug.

Due to the symmetry of the geometry by *v*, we focus on the left half and locate four positions (*i* to *iv*) where field strength changes. The first point *i* is at the interface between the air and the susceptibility-matching material, where an abrupt decrease of the field strength is induced. It is clear that a 0.01 m susceptibility-matching plug is sufficient to eliminate the inhomogeneity brought by the interface change. The second point *ii* shows a turbulence of the field due to the metal striplines covering the channel. Points *iii* and *iv* indicate the Lenz lens side-wing, showing a bump on the B_0 curve. The static field strength inside

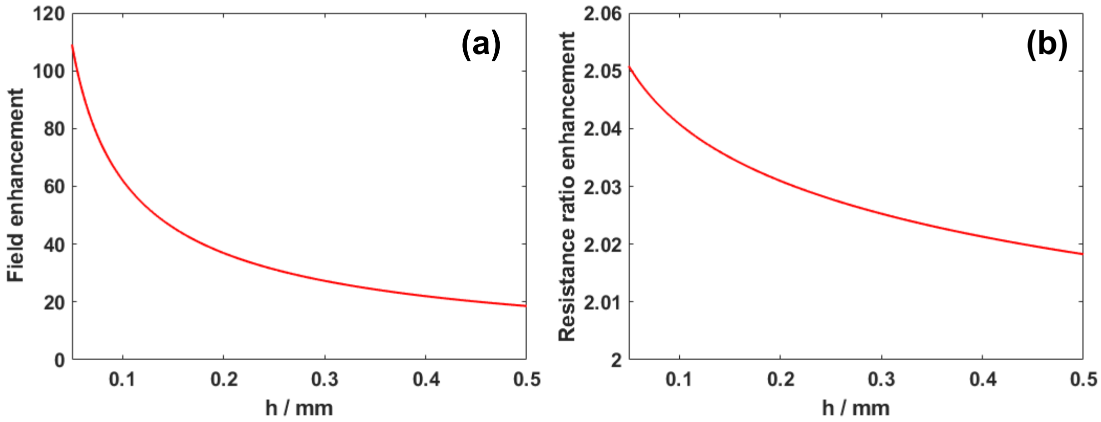

Figure S1: Parameter change vs. vs. stripling spacing h. (a) B_1 magnification at unit current. (b) terminal resistance.

the Lenz lens is of high homogeneity, within a few *ppb*. The inhomogeneities at the edges are caused by the loop wings.

An enhanced RF field generated by the S3L is present along the purple-tinted length section. Through a simulated nutation spectrum shown in Fig. S2 at this region, $A_{450^\circ}/A_{90^\circ}$ is around 85%, which indicates a good RF field homogeneity. The computed value matches the measured value, as reported in the main text.

In an ideal case, the measured sample should not exceed the conjunction point *iii* from the center to avoid the field disturbance. Further study can be addressed on how to fill the sample into the homogeneous region preciously.

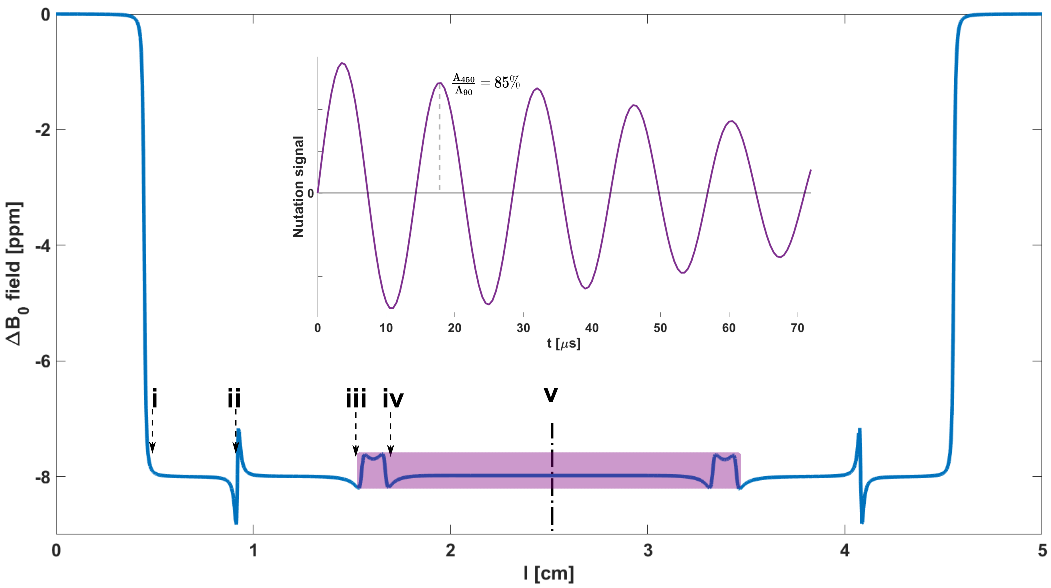

Figure S2: B_0-field strength along the centreline of the chip, intersecting the sample channel and the *S3L*. The insert shows the theoretical proton nutation spectrum envelope, resulting from the magnetic field profile corresponding to the sample region as highligted in purple.

References

[1] Jacob Bart, Ard J Kolkman, Anna Jo Oosthoek-de Vries, Kaspar Koch, Pieter J Nieuwland, Hans Janssen, Jan van Bentum, Kirsten AM Ampt, Floris PJT Rutjes, Sybren S Wijmenga, et al. A microfluidic high-resolution NMR flow probe. *Journal of the American Chemical Society*, 131(14):5014–5015, 2009.

[2] Bart, J. and Janssen, J. W. G. and van Bentum, P. J. M. and Kentgens, A. P. M. and Gardeniers, Johannes GE. Optimization of stripline-based microfluidic chips for high-resolution NMR. *Journal of magnetic resonance*, 201(2):175–185, 2009.

[3] F Bonetto, E Anoardo, and M Polello. Saddle coils for uniform static magnetic field generation in NMR experiments. *Concepts in Magnetic Resonance Part B: Magnetic Resonance Engineering: An Educational Journal*, 29(1):9–19, 2006.

[4] Ying Chen, Hardeep S Mehta, Mark C Butler, Eric D Walter, Patrick N Reardon, Ryan S Renslow, Karl T Mueller, and Nancy M Washton. High-resolution microstrip NMR detectors for subnanoliter samples. *Physical Chemistry Chemical Physics*, 19(41):28163–28174, 2017.

[5] Graeme Finch, Ali Yilmaz, and Marcel Utz. An optimised detector for in-situ high-resolution NMR in microfluidic devices. *Journal of Magnetic Resonance*, 262:73–80, 2016.

[6] Mazin Jouda, Robert Kamberger, Jochen Leupold, Nils Spengler, Jürgen Hennig, Oliver Gruschke, and Jan G Korvink. A comparison of Lenz lenses and LC resonators for NMR signal enhancement. *Concepts in Magnetic Resonance Part B: Magnetic Resonance Engineering*, 47(3):e21357, 2017.

[7] Hans Georg Krojanski, Jörg Lambert, Yilmaz Gerikalan, Dieter Suter, and Roland Hergenröder. Microslot NMR probe for metabolomics studies. *Analytical chemistry*, 80(22):8668–8672, 2008.

[8] Anna Jo Oosthoek-de Vries, Jacob Bart, Roald M Tiggelaar, Johannes WG Janssen, P Jan M van Bentum, Han JGE Gardeniers, and Arno P M Kentgens. Continuous flow 1H and 13C NMR spectroscopy in microfluidic stripline NMR chips. *Analytical chemistry*, 89(4):2296–2303, 2017.

[9] Edward Bennett Rosa. *The self and mutual inductances of linear conductors*. Number 80. US Department of Commerce and Labor, Bureau of Standards, 1908.

[10] Herbert Ryan, Suk-Heung Song, Anja Zaß, Jan Korvink, and Marcel Utz. Contactless NMR spectroscopy on a chip. *Analytical chemistry*, 84(8):3696–3702, 2012.

[11] Manvendra Sharma and Marcel Utz. Modular transmission line probes for microfluidic nuclear magnetic resonance spectroscopy and imaging. *Journal of Magnetic Resonance*, 303:75–81, 2019.

[12] Eric G. Sorte, Nathan A. Banek, Michael J. Wagner, Todd M. Alam, and Yu Ye J. Tong. In Situ Stripline Electrochemical NMR for Batteries. *ChemElectroChem*, 5(17):2336–2340, 2018.

[13] P. J. M. Van Bentum, J. W. G. Janssen, A. P. M. Kentgens, J. Bart, and J. G E Gardeniers. Stripline probes for nuclear magnetic resonance. *Journal of Magnetic Resonance*, 189(1):104–113, 2007.
